# Hyperglycemia in intensive care identifies patients at high risk of developing diabetes: preliminary data of a multicentre study

**DOI:** 10.1186/cc12395

**Published:** 2013-03-19

**Authors:** E Lukic, I Gornik, M Grgic-Medic, D Milicic, V Vegar, V Ivancan, M Peric, V Gasparovic, I Pavlic Renar

**Affiliations:** 1 University Hospital Centre Zagreb, Croatia

## Introduction

A recent study showed that hyperglycaemia (blood glucose ≥7.8 mmol/l) in nondiabetic patients hospitalised in a medical ICU is associated with increased risk of diabetes [1]. We investigated a large mixed ICU population to confirm these results.

## Methods

This study retrospectively included patients with negative history of diabetes admitted to ICUs during the year 2007. We excluded patients receiving steroids, with newly diagnosed diabetes and those with end-stage disease. Patients were followed-up 5 years after index admission. Diagnosis of diabetes within 63 months from the index admission was presumed as revealing DM at inclusion, which excluded the patient. Patients who were taking glucocorticoids during the follow-up period were excluded. The rest of the patients, if consented, were assessed for their diabetic status with a single HbA1c measurement.

## Results

A total of 2,696 patients were included in the study: 1,441 medical and 1,255 surgical, hyperglycaemia (≥7.8 mmol/l) was registered in 1,419 (52%). During the 5 years from index admission 409 died and 38 were taking glucocorticoids, and 10 refused participation. Diabetes was diagnosed in 189 patients during the 63months after index admission. The remaining cohort of 2,320 patients had 1,169 patients who were hyperglycaemic during hospitalisation. Diabetes was already diagnosed in 132 patients, of whom 112 were hyperglycaemic in the ICU; prediabetes was already diagnosed in 121 patients, of whom 83 had ICU hyperglycaemia. Since 125 patients (55 hyperglycaemic) refused HbA1c testing, it was performed on 1,852 patients and revealed diabetes in 136 patients (107 had hyperglycaemia) and prediabetes in 103 patients (75 were hyperglycaemic in ICU). Overall, patients who had hyperglycaemia had 19.8% cumulative 5-year incidence of diabetes and 14.3% of prediabetes. Nonhyperglycaemic ICU patients had cumulative incidences of 4.5% and 6.6%, respectively. Relative risk for developing diabetes during 5 years after ICU admission is 4.2 (95% CI = 3.2 to 5.9) for patients who did have hyperglycaemia.

## Conclusion

The results of this study confirm in a large mixed medical/ surgical ICU population that hyperglycaemia occurring in severe illness or the perioperative period is associated with increased risk of developing diabetes. Suggesting lifestyle changes to reduce the risk and regular follow-up should be implemented at least for these patients.
